# Mutation c.256_257delAA in *RAG1* Gene in Polish Children with Severe Combined Immunodeficiency: Diversity of Clinical Manifestations

**DOI:** 10.1007/s00005-016-0447-1

**Published:** 2017-01-12

**Authors:** Anna Szaflarska, Magdalena Rutkowska-Zapała, Monika Kotula, Anna Gruca, Agnieszka Grabowska, Marzena Lenart, Marta Surman, Elżbieta Trzyna, Anna Mordel, Anna Pituch-Noworolska, Maciej Siedlar

**Affiliations:** 1 Department of Clinical Immunology, Institute of Pediatrics, Jagiellonian University Medical College, Wielicka 265, 30-663 Krakow, Poland; 2 Department of Clinical Immunology, University Children’s Hospital, Wielicka 265, Krakow, Poland; 3 Department of Medical Genetics, Institute of Pediatrics, Jagiellonian University Medical College, Wielicka 265, Krakow, Poland; 4 Department of Medical Genetics, University Children’s Hospital, Wielicka 265, Krakow, Poland; 5 Department of Transplantation, Institute of Pediatrics, Jagiellonian University Medical College, Wielicka 265, Krakow, Poland; 6 Department of Transplantation, University Children’s Hospital, Wielicka 265, Krakow, Poland

**Keywords:** *RAG1/2* genes, Severe combined immunodeficiency, Omenn syndrome

## Abstract

Mutations in *RAG1* gene may result in different types of severe combined immunodeficiencies. In this study, we compare clinical symptoms and laboratory findings in four children with identical mutation in *RAG1* gene. All of analyzed patients presented symptoms of severe combined immunodeficiencies associated or not with Omenn syndrome (OS) features. In our patients two different types of variants in *RAG1* gene were detected. The first of the mutation was the deletion of AA dinucleotide at position c.256_257 (p.Lys86ValfsTer33), the second gene variant was substitution c.2867T>C (p.Ile956Thr). In Patient 1 we detected that compound heterozygous mutations involved both of the mentioned variants. Whereas, in Patients 2, 3 and 4, we confirmed the presence of the dinucleotide deletion but in a homozygous state. In all described patients, sequence analysis of *RAG2* gene did not reveal any nucleotide changes. Our data show that mutation c.256_257delAA in *RAG1* gene seems to occur quite frequently in the polish patients with severe combined immunodeficiency and may result in classical OS as well as in severe combined immunodeficiency without clinical and laboratory features of OS when occurred in homozygous state. The same mutation but in heterozygous state, in combination with other mutation in *RAG1* gene, may result in incomplete OS.

## Introduction

The recombination-activating gene (RAG) 1 and RAG2 proteins, encoded by two genes *RAG1* and *RAG2*, respectively, on chromosome 11p23, are lymphoid-specific components of the complex of enzymes termed the V(D)J recombinases. They play a pivotal role in the process of re-arrangement of the variable (V), diversity (D) and joining (J) segments during the development of the immunoglobulin and T-cell receptors. Numerous mutations at *RAG1* and *RAG2* genes can lead to a range of phenotypes characterized by various clinical spectrum (Niehues et al. 2010). Null mutations, associated with less than 1% of wild-type recombination activity typically result in the severe combined immunodeficiency (SCID) lacking T and B cells (T^−^B^−^). However, hypomorphic mutations associated with residual RAGs activity can result in different immunologic phenotypes. These include: Omenn syndrome (OS), characterized by erythroderma, lymphadenopathy, eosinophilia, increased serum IgE levels, and the presence of oligoclonal T cells; “leaky” SCID, with varying numbers of T and B cells but without the typical features of OS; SCID with expansion of γδ T lymphocytes; delayed-onset combined immune deficiency with granuloma and/or autoimmunity; and in a single case of idiopathic CD4^+^ T-cell lymphopenia, presenting with extensive chickenpox and recurrent pneumonia (Ijspeert et al. 2014; Kuijpers et al. 2011; Lee et al. 2014; Niehues et al. 2010; Omenn 1965).

In this study, we analyzed inherited immunodeficiencies in four children, two girls and two related boys, caused by mutations in *RAG1* gene. In the first girl (Patient 1) we identified compound heterozygous mutations c.256_257delAA (p.K86VfsX33) and c.2867T>C (p.I956T). The same gene variant, namely c.256_257delAA, was observed in the other three children (Patients 2, 3 and 4) but at the homozygous state. Interestingly, each of these patients represents different clinical manifestations: from the incomplete OS (Patient 1) through SCID without OS features (Patient 2) to typical OS (Patient 3 and 4).

## Patients and Methods

### Patients

#### Patient 1

**Fig. 1:**
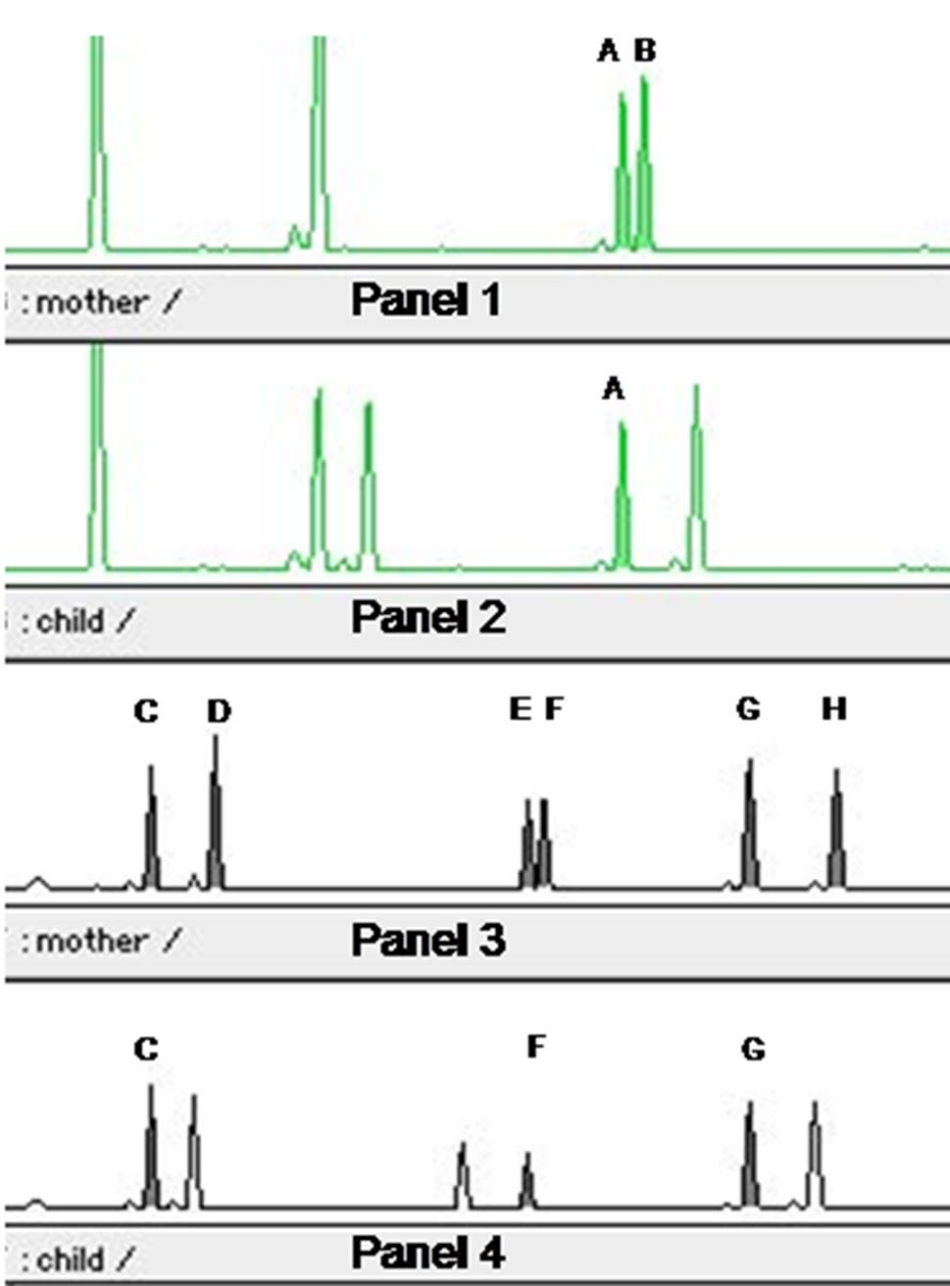
Distribution of peaks representing amplification of STR loci in Patient 1 and his mother (*A*, *B* D21S11; *C*, *D* D19S433; *E*, *F* TH01; *G*, *H* FGA). Informative loci used to determine maternal-child’s chimerism are represented by *filled peaks*. In the *panel 2* and *4*: there are maternal peaks *A*, *C*, *F*, *G* observed in child’s sample but there are no other maternal peaks (*B*, *D*, *E*, *H*) characteristic to the particular STR loci

The girl weighting 3200 g was delivered spontaneously at 38-week of gestation to a gravida 1 para 1 mother with Apgar score of 10 at first minute. Her young, unconsanguineous parents were healthy, without any symptoms of immunodeficiency. She was vaccinated with BCG and Engerix B without complications. The patient was admitted to the hospital at the age of 31 days. Physical examination revealed erythrodermia, no hepatosplenomegaly and no lymphadenopathy. Apart from erythematous rash and exfoliation of the whole body skin, the signs of bilateral pneumonia were noted. In blood morphology elevated number of eosinophils was detected (Table 1). Liver and renal function tests were normal. Tests for cytomegalovirus (CMV) infection were positive (317,913 copy/ml in blood, 94,000 copy/ml in cerebrospinal fluid (BCG), polymerase chain reaction (PCR) method). *Staphylococcus epidermidis MRSE, MLS* and *Enterococcus faecalis* were cultured in the blood. Immunological tests revealed normal levels of serum immunoglobulins: IgG, IgA, IgM and high level of IgE. Flow cytometric analysis showed decreased absolute number of T cells (both CD4^+^ and CD8^+^ subpopulations), increased number of activated lymphocytes T (CD3^+^/HLA^−^DR^+^). The vast majority of T cells were CD45RO^+^. B lymphocytes were absent. Percentage and absolute number of NK cells were within the normal range. The response of lymphocytes to stimulation of mitogens in proliferation test was normal (Table 2). Maternal T-cell engraftment was excluded due to negative results of mother T lymphocytes chimerism test (Fig. 1). On the basis of clinical and laboratory findings OS was the putative diagnosis. Therefore, the *RAG1* and *RAG2* genes were selected for sequence analysis. The child was bottle-fed but despite high protein diet (2.7 g/kg/24 h) the protein levels were still below the normal ranges. On admission she received two antibiotics. Co-trimoxazole, ketoconazole, ganciclovir sodium with foscarnet, methylprednisolone hemisuccinate (0.4 mg/kg) and IVIG substitution (every 3 weeks in dose 500 mg/kg) were included into therapy schedule. Shortly after the compatible unrelated donor was found, the child was transferred to Bone Marrow Transplantation Center.

**Table 1:** The number of white blood cells (WBC) and their population at first examination in four our patients with *RAG1* mutations

WBC (cells/μl × 10^3^)	Patient 1		Patient 2 (age-matched normal values^a^)		Patient 3		Patient 4		Age-matched normal values for Patients 1, 3 and 4	
	21.9		9.5 (*5.0*–*14.8*)		28		19.6		*6.0–16.3*	
	% of WBC	cells/μl × 10^3^	% of WBC	cells/μl × 10^3^	% of WBC	cells/μl × 10^3^	% of WBC	cells/μl × 10^3^	% of WBC	cells/μl × 10^3^
Lymphocytes	43.3	9.5	1.5 (*11*–*58*)	0.14 (*0.55*–*8.58*)	56	15.68	8	1.57	*21.3*–*69.8*	*0.9–5.2*
Neutrophils	15.9	3.5	8.9 (*30.4*–*78.6*)	7.71 (*1.52*–*11.63*)	6	1.68	26	5.09	*13.6*–*58.9*	*2.5*–*6.5*
Eosinophils	26.2	5.7	0.0 (*0*–*4.3*)	0.0 (*0*–*0.64*)	35	9.8	62	12.15	*0*–*5.7*	*0*–*0.6*
Monocytes	13.8	3.0	10.9 (*3.3*–*13.9*)	1.04 (*0.16*–*2.06*)	3	0.84	4	0.78	*2.4*–*19.6*	*0.1*–*1*

^a^Age-matched normal values for Patient 2 (shown in brackets) were presented together with his results and correspond to patient’ age that differ from the remaining patients (age-matched normal values for Patient 1, 3 and 4 were shown in the last column)

**Table 2:** Humoral and cellular immunity parameters at first examination in patients with RAG1 mutations

Parameter	Patient 1	Patient 2 (age-matched normal values^a^)	Patient 3	Patient 4	Age-matched normal values for Patients 1, 3 and 4 (Piątosa et al. 2010)
Serum immunoglobulins(g/l)					
IgG	8.45	4.80 (*3.83*–*10.1*)	5.67	9.59	6.80–15.30
IgA	0.09	0.60 (*0.17*–*1.05*)	<0.06	0.08	0.00–0.05
IgM	0.34	1.39 (*0.29*–*1.50*)	0.34	0.73	0.00–0.17
IgE total (IU/ml)	3640	<16.4 (*0.0*–*60.0*)	32,000	38,000	0.00–1.50

Lymphocyte subpopulations	% of lymph	cells/μl	% of lymph	cells/μl	% of lymph	cells/μl	% of lymph	cells/μl	% of lymph	cells/μl
CD3	59	1575^c^	20 (*53*–*75*)	119 (*2100*–*6200*)	90	9000	70	1190	53–84	2500–5500
CD4	57	1521	15 (*32*–*51*)	89 (*1300*–*3400*)	38	3800	58	986	35–64	1600–4000
CD8	2	53	3 (*14*–*30*)	18 (*620*–*2000*)	46	4600	11	187	12–28	560–1700
CD19	1	27	27 (*16*–*35*)	27 (*720*–*2600*)	1	4	2	78	6–32	300–2000
CD22	0.17	5	nd	nd	nd	nd	nd	nd	6–32	300–2000
CD20	0.19	5	nd	nd	nd	nd	nd	nd	6–32	300–2000
HLA-DR	61	1628	6 (*5*–*8*)	36 (*135*–*232*)	76	6840	56	1638	5–8	135–232
CD3/CD45RO	99	2642^c^	nd	nd	99.8	8982	98.6	1173	4.9–15.1	30–180
NK cells CD3^−^CD16^+^CD56^+^	36	961	50 (*3*–*15*)	298 (*180*–*920*)	3	300	12	204	4–18	170–1100

Lymphocyte proliferation (cpm)					Control group^b^
Medium	955	780	768	616	1367±1110
PHA	18,143	3527	7722	2026	51,899±2969
Anti**-**CD3	10,655	3099	8477	655	48,785±24,025
PWM	9644	2243	8660	2033	50,255±21,594

*PHA* phytohaemagglutinin, *PWM* pokeweed mitogen, *nd* not determined

^a^As in Table 1

^b^Control group: healthy 34 children (18 girls and 16 boys), the medium age 2.1 ± 1.1 years

^c^Analysis performed in separate days

#### Patient 2

The girl weighting 3180 g was delivered spontaneously at 39-week of gestation to a gravida 2 para 2 mother with Apgar score of 9 at first minute. Her young unconsanguineous parents and her elder brother were healthy. She was vaccinated with BCG and Engerix B without complications. From birth she presented failure to thrive. Periodic diarrheas were observed. She had pneumonias in the third and sixth month of her life, treated in hospital. In 18 month of life she developed severe bilateral lobar pneumonia with respiratory insufficiency (from this time—PCR tests for respiratory syncytial virus (RSV) were positive) and candidiasis of gastrointestinal tract. In blood morphology lymphopenia was detected (Table 1). Immunological tests revealed normal levels of serum immunoglobulins: IgG, IgA, IgM and IgE. Flow cytometric analysis showed significantly decreased absolute number of T cells (both CD4^+^ and CD8^+^ subpopulations) and B cells, the number of activated lymphocytes T (CD3^+^/HLA^−^DR^+^) was slightly decreased. Percentage and absolute number of NK cells were within the normal range. The response of lymphocytes to stimulation of mitogens in proliferation tests was abnormal (Table 2). On the basis of the clinical course and the results of immunological tests, the diagnosis of T^−^B^−^NK^+^ SCID was estabilished, which was proved in genetic analysis. She was treated with numerous antibiotics, Co-trimoxazole and ketoconazole. She received also IVIG substitution (every 3 weeks in dose 500 mg/kg). The girl was transferred to Bone Marrow Transplantation Center. She has died after unsuccessful hematopoietic stem cell transplantation (HSCT) because of respiratory insufficiency.

#### Patient 3 and Patient 4

Patient 3 and 4, from the same extended family and with classical clinical and immunological features of OS, were described previously (Ijspeert et al. 2014; Szaflarska et al. 2009).

## Methods

### Immunological Assays

Concentration of serum immunoglobulin level (IgG, IgA, IgM, IgE) was determined by a nephelometry (BNII nephelometer, Dade-Behring, Deerfield, IL, USA; antisera from Behring) during routine laboratory tests. The percentage and absolute numbers of particular subpopulation of lymphocytes were determined by flow cytometry using the lyse–no wash whole blood MultiTEST procedure and reagents (BD Biosciences). Samples were analyzed on FACS-Calibur Becton–Dickinson (BD) flow cytometer. Lymphocyte response to antigen/mitogen stimulation was measured in vitro using 3H-thymidine incorporation assay.

### Molecular Analysis

#### DNA Isolation

Genomic DNA was isolated using QIAmp DNA Mini Kit (Qiagen, Hilden, Germany) from peripheral blood drawn into tubes containing ethylenediaminetetraacetic acid (EDTA) as anticoagulant. DNA concentrations and quality were measured using Quawell Q5000 UV–vis spectrophotometer.

#### Sequencing the *RAG1* and *RAG2* Gene

Coding sequences of the studied genes were amplified by PCR, which was performed using 100 ng spectrophotometrically quantified DNA, 0.3 units of AmpliTaq Gold polymerase (Applied Biosystems, Foster City, CA, USA), 0.5 mM of each dNTP, 2.5 mM MgCl_2_, and 0.5 µM of each primers (Genomed, Poland) specific to the second and third exon of the *RAG1* and *RAG2* gene, respectively. The reaction volume was 20 µl. Primer sequences for amplification and sequencing reactions are listed in Table 3. The following PCR cycling conditions were used: 1 cycle of 95 °C for 10 min, 30 s at 95 °C, 30 s at 58 °C and 1 min at 72 °C, repeated for 35 cycles, followed by 72 °C for 10 min. The PCR products were purified with a ExoSAP-IT For PCR Product Cleanup (Affymetrix) according to manufacturer’s instructions. Then, Sanger sequencing PCR reaction was performed using Big Dye Terminator v3.1 Cycle Sequencing Kit (Applied Biosystems), exon-specific primer and following cycling conditions: an initial denaturation at 95 °C for 1 min followed by 25 cycles, with 1 cycle consisting of 10 s at 95 °C, 5 s at 50 °C, and 4 min at 60 °C. PCR products were purified by ethanol/EDTA precipitation and dissolved in Hi-Di Formamide (Applied Biosystems), denatured at 95 °C for 3 min, and run on the Applied Biosystems 3500 Genetic Analyzer with the use of POP-7 polymer, 50 cm capillary, default run parameters, and analyzed with the Sequencing Analysis software v5.4 (Applied Biosystems). For each exon the bidirectional sequence analysis was performed. The DNA sequencing results were aligned to the *RAG1* and *RAG2* gene sequences available in the Ensembl database (reference sequences of *RAG1* and *RAG2* gene: ENSG00000166349 and ENSG00000175097, respectively; the numbering of exons and mutation nomenclature in *RAG1* gene according to the transcript ENST00000299440).

**Table 3:** Primers for PCR amplification of *RAG1* and *RAG2* gene

Exon No.	Forward primer (5′ → 3′)	Reverse primer (5′ → 3′)
*RAG1*		
2a	TCTATGATCAGCACCTAACATGA	TAAACCTCACATGGGGCACT
2b	AGTTCTGCCATAACTGCTGG	ACTGACTGCAGCTGAGGAAG
2c	GTGAAGTCCGTGTGCATGAC	AGGTTCTCAGCATGGCTTCT
2d	CCTGCTAAAGAGTGCCCAGA	GCCTGAGGGTTCATGGTAAA
2e	TTATTGAGAGGGATGGCTCC	ATGACAGCAGATGACCTCCT
*RAG2*		
3a	GGTTCTGTGGCTCTTTACTG	TTGGCAAGTGAATGTCCTCC
3b	TGAATTTGGGTGTGCTACATC	CTTGCTATCTCCACATGCTC
3c	GCTACTGGATTACATGCTGCC	CCTCGATGATTATTACTGCTTCTG

Results of sequence analysis of *RAG1* and *RAG2* gene in Patients 2, 3 and 4 were provided by Erasmus Medical Center in Rotterdam.

#### Maternal-Child’s Chimerism Analysis

Chimerism analysis was performed only in Patient 1. DNA was isolated from the blood of the Patient 1 using DNA isolation kit (Qiagen) and amplified with the use of AmpFlstr SGM Plus kit (Applied Biosystems). Afterwards, fluorescently labeled PCR products were capillary electrophoresed on sequencing apparatus ABI Prism 310 with the use of POP-4 polymer and 45 cm capillary. Resulting signals were analyzed with Gene Scan v.3.1.2 software.

## Results

**Fig. 2:**
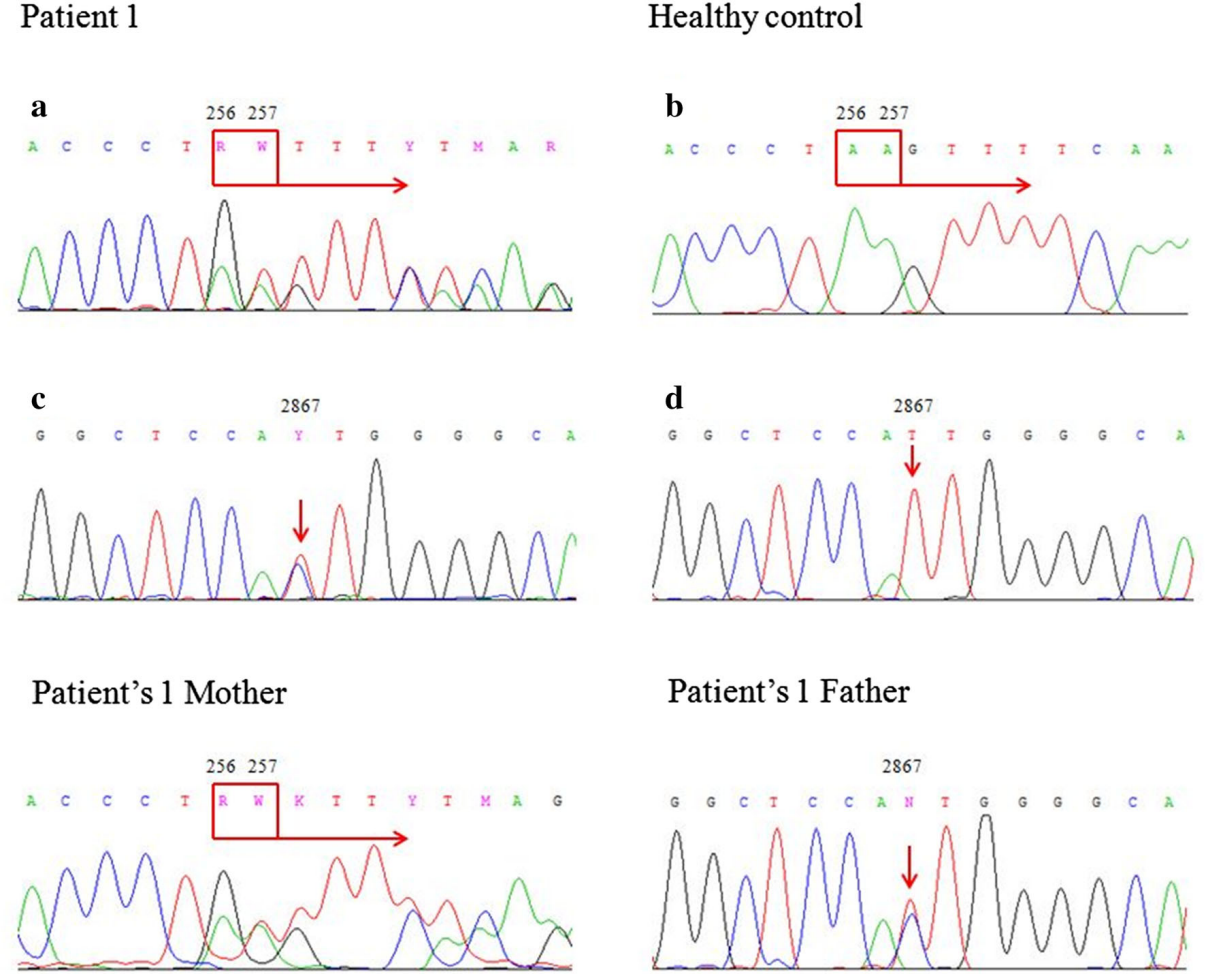
Electropherograms of the fragment of the *RAG1* gene’s second exon of the Patient 1, showing two types of indicated gene variants: deletion c.256_257delAA, resulting in frameshift variant: p.Lys86ValfsTer33 (**a**) and T to C substitution at the position c.2867, resulting in amino acid change of isoleucine to threonine (p.Ile956Thr) (**c**). Next to the changes observed in the Patient 1, the correct sequences of the analyzed fragments of the gene in a healthy control were shown: nucleotides 256 and 257 (**b**) and nucleotide 2867 (**d**), respectively. The other two electropherograms of the *RAG1* gene of the patient’s parents, confirmed carriers of the nucleotide variants observed in the child: a deletion c.256_257delAA detected in the mother and substitution at the position 2867 observed in the father. Positions of the mentioned nucleotides are indicated by the *square with an arrow* or by the *arrows*. The sequence fragments were displayed by Chromas Lite software

Our studies concentrated on mutations on the translated sequences of *RAG1* and *RAG2* genes, including second and third exon, respectively. In all described patients, sequence analysis of *RAG2* gene did not reveal any nucleotide changes, either in the form of mutations or polymorphisms, in comparison to control subjects and reference sequences available in Ensembl database. Within the *RAG1* gene, two different types of variants were detected. Both of them were observed in Patient 1. The first of the mutations was the heterozygous deletion of AA dinucleotide at position c.256_257 leading to a frameshift followed by a premature stop codon (p.Lys86ValfsTer33). The second gene variant found in this patient was heterozygous missense mutation c.2867T>C resulting in p.Ile956Thr amino acid substitution. Sequence analysis of *RAG1* gene in patient’s parents was also performed in order to determine the inheritance of the detected variants. The first gene variant detected in patient (c.256_257delAA) was found in the mother, the second (c.2867T>C) in the father. The heterozygous state of both healthy parents was confirmed. Carrier state of parents indicates that the child is likely the compound heterozygote for two different *RAG1* mutations in trans position, one in each allele. Patient’s genotype was as follows: ENST00000299440: c.(256_257delAA); (2867T>C) (the nomenclature according to Human Genome Variation Society recommendations). Results of DNA sequencing are shown in the Fig. 2. Sanger sequencing of *RAG1* gene performed in Patients 2, 3 and 4 confirmed the presence of the first of the mentioned gene variants, dinucleotide deletion at position c.256_257 but in a homozygous state. Their genotypes were: ENST00000299440: c.(256_257delAA); (256_257delAA).

## Discussion

In this study, we described patients with SCID in whom the same mutations (c.256_257delAA) in *RAG1* gene were detected, both in the heterozygous (Patient 1) or homozygous state (Patient 2, 3 and 4). All of the patients showed clinical features of SCID (severe infections, failure to thrive), with or without OS symptoms (Table 4). Phenotype presented in Patient 1 was similar to incomplete OS (without lymphadenopathy and hepatosplenomegaly); however, the T-cell oligoclonality was not tested. Clinical symptoms in this child comprised early onset erythrodermia with desquamation and concomitant severe protracted CMV infection. The results of laboratory test revealed elevated level of total IgE, eosinophilia, increased number of activated T cells, low number of peripheral B cells and expansion of autologous T cells CD45^+^RO^+^ which were the hallmarks of OS. In Patient 1 compound heterozygous mutations in *RAG1* gene were found. Both of the mentioned variants were reported previously as the recessive alleles. The first mutation, homozygous dinucleotide deletion c.256_257delAA have been found to be hypomorphic and lead to translation starting from an alternative initiation site (alternative ATG), at a second methionine codon downstream from the deletion (de Villartay et al. 2005). This reinitiation of the translation results in a partially functional N-terminally truncated RAG1 protein. The second mutation indicated in our Patient 1 was described for the first time by Sobacchi et al. (2006) with clinical symptoms suggesting OS. In contrast to previous observation, in our study both observed gene variants were heterozygous. According to our best knowledge, compound heterozygous mutations observed in Patient 1 have not been described so far. Sequence studies performed in patient’s parents confirmed the carriers of the mutations by both of them. Despite of carrying the mutation, no clinical symptoms of immunodeficiency were observed in parents. Therefore, it can be assumed that compound heterozygosity of this two pathogenic variants causes an effect of the SCID observed in our patient under mask of phenotypic incomplete OS manifestation. The heterozygosity state for either mutation probably results in partial RAG1 deficiency, each of them reduce the level of gene activity.

**Table 4:** Comparison of the clinical symptoms in children with mutations in *RAG1* gene

	Compound heterozygous mutations: c.256_257delAA and c.2867T>C (Patient 1)	Homozygous deletion c.256_257delAA (Patient 2)	Homozygous deletion c.256_257delAA (Patient 3)	Homozygous deletion c.256_257delAA (Patient 4)
Hepatosplenomegaly	−	−	+	+
Lymphadenopathy	−	−	+	+
Early onset erythrodermia	+	−	+	+
Presence of activated T cells	+	−	+	+
Oligoclonal αβ-T cell repertoire	nd	nd	Oligoclonal	Clonal
Hypereosinophilia	+	−	+	+
Elevated serum IgE	+	−	+	+
CMV infection	+	−	+	−
Pneumonia	+	+	+	+
Protracted diarrhea	−	+	+	−
Failure to thrive	+	+	+	+
Cytopenias	−	+ (Thrombocytopenia)	+ (Neutropenia)	−

*Nd* not determined

Patients 2 had no clinical and laboratory features typical for OS. Before reaching the age of 18 months he had had no life threatening infections. At this time he developed severe and prolonged RSV infection with respiratory insufficiency. Sequence analysis revealed in this patient homozygous deletion c.256_257delAA, which was exactly the same as in Patients 3 and 4. However, Patients 3 and 4 presented classical OS.

Summarizing, our data show that described mutation c.256_257delAA in *RAG1* gene seems to occur quite frequently in Polish SCID children diagnosed in our Center: four cases per ten patients with molecular diagnosis of SCID in the past 6 years. If the T-cell receptor excision circles analysis had been performed, all our patients might have been diagnosed with SCID much earlier. Performing that assay in Patient 2 could have prompted the decision of HSCT before developing life threatening infections. Unfortunately, such analysis is not included in newborn screening panel in Poland.

In conclusion, our results are similar to those presented earlier (Corneo et al. 2001; Ijspeert et al. 2014; Martinez-Martinez et al. 2012; Pasic et al. 2009; Sharapova et al. 2016) and indicated that specific mutations in *RAG1* gene are associated with phenotypic heterogeneity and do not predict the clinical phenotype of a patient. The authors of mentioned studies postulate that this fact is most likely concerned with the type of antigenic stimulation and residual recombinase activity. However, in our opinion it may be also the effect of polygenic penetrance of specific mutations which could be modified by the presence or absence of additional undetected variants in the form of mutations as well as polymorphisms at other gene loci, even in gene regions which are still not connected to SCID phenotype. Useful method for the analysis of wide range of gene panels in such patients could be the next generation sequencing techniques.
